# Systematic review of non-surgical etiologies of nasal necrosis: evidence from case reports and case series

**DOI:** 10.1007/s00405-026-10406-6

**Published:** 2026-07-27

**Authors:** Marie Line Chedid, Rima Mohammad Achraf Hariri, Talia El Chaer, Veronica Andrade Murillo, Nancy Emmanuel

**Affiliations:** 1 Faculty of Medicine, Medical University of Łódż, Łódż, Poland; 2 Lebanese University Faculty of Medical Sciences, Hadath, Lebanon; 3 University of Balamand, Tripoli, Lebanon; 4 Universidad Católica de Santiago de Guayaquil, Guayaquil, Ecuador; 5 Department of Dermatology, Hospital Das Clínicas of the Faculty of Medicine, University of Sao Paulo, R. Dr. Eneas Carvalho de Aguiar, 250 - Cerqueira César, São Paulo, SP 05403-010 Brazil

**Keywords:** Nasal necrosis, Nasal tip ischemia, Cutaneous necrosis, Non-surgical management, Infectious etiologies, Drug induced necrosis

## Abstract

**Purpose:**

Nasal necrosis is a rare but potentially devastating manifestation of diverse conditions, including fulminant infection, immune-mediated vasculopathy, drug toxicity, neoplasia and device-related pressure injury. Surgical intervention is often prioritized; however, a subset of patients is managed non-surgically and evidence-based guidance remains limited. The objective is to systematically synthesize non-surgical etiologies, clinical patterns, diagnostic workup, and management strategies reported in cases of nasal necrosis.

**Methods:**

A systematic search of PubMed, Scopus and ScienceDirect from inception to December 7, 2025 identified case reports and case series involving human patients with nasal skin or soft-tissue necrosis not primarily attributable to surgery or cosmetic procedures. Non-human studies, systematic reviews and non english records were excluded. Data on demographics, etiology, presentation, investigations, management and outcomes were extracted. Methodological quality was appraised using Joanna Briggs Institute (JBI) tools.

**Results:**

Thirty-nine publications comprising 45 cases were included. Infectious etiologies were most frequent (44.4%), followed by mechanical or traumatic (20.0%), immune-mediated (13.3%), drug-induced (13.3%), neoplastic (6.7%), and congenital causes (2.2%). Isolated nasal involvement occurred in 57.8% of cases. Non-surgical management included antimicrobials, corticosteroids, immunosuppressive agents, anticoagulation, wound care, and adjunctive hyperbaric oxygen therapy, with better outcomes in cases where the underlying cause was identified early.

**Conclusion:**

Nasal necrosis represents a heterogeneous clinicopathologic endpoint in which prompt etiologic diagnosis is critical for effective, organ-preserving dermatologic management.

## Introduction

Nasal and facial skin necrosis is an uncommon clinical finding resulting from a wide spectrum of etiologies, including inflammatory, mechanical, infectious, and substance-abuse related conditions, necessitating a comprehensive differential diagnosis [1]. This rare condition presents significant challenges in clinical management and can lead to serious health consequences such as physical impairment, cosmetic deformities, and, in severe cases, systemic illness [2–4].

Surgical intervention is often considered the first-line treatment to restore tissue viability and prevent further damage. However, there exists a subset of patients that require a less invasive approach, especially those with immune-mediated etiologies, contraindications to surgery and limited tissue involvement.

However, standardized guidelines for the dermatological treatment of nasal necrosis remain absent. Consequently, clinicians are often left without a standardized evidence based framework to guide diagnostic evaluation and treatment decisions. In order to address this gap, we therefore conducted a systematic review of published case reports and case series excluding surgical or cosmetic procedures with the aim to describe the etiologies, clinical patterns and non surgical management strategies, providing the most comprehensive synthesis available to date and supporting the development of informed dermatologic guidance.

**Fig. 1:**
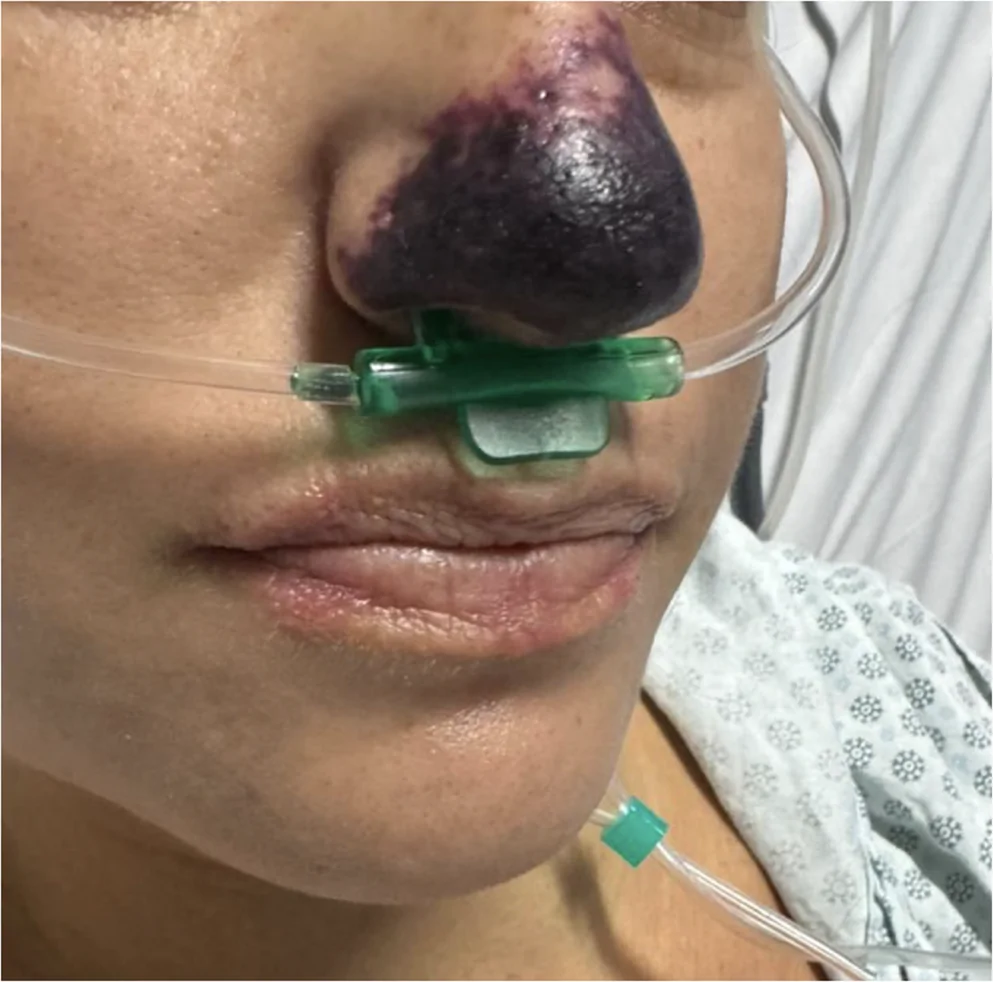
Clinical presentation of nasal tip necrosis

A representative clinical example of nasal tip necrosis is shown in (Fig. 1).

## Methods

This review was conducted in accordance with PRISMA 2020 guidelines and included case reports and case series characterizing nasal necrosis etiologies and management as a cross-disciplinary problem addressed by different medical professionals and having underlying medical disease of vast variety. The review focused on non-surgical causes of nasal necrosis and synthesized published case reports and case series.

### Eligibility criteria (PICOS)

Population (P): Human population of any age and race, presenting with nasal skin or soft tissue necrosis. Intervention (I): Assessment of cases of nasal necrosis cases of non-surgical etiologies and management approaches for nasal necrosis. Comparison (C) : No formal comparator was used; cases were analyzed descriptively based on the associated signs and symptoms as well as laboratory and imaging results. Outcome (O): Underlying etiology, clinical presentation, diagnosis of the underlying cause of nasal necrosis and treatment. Study design (S): Case reports case-series, we excluded surgical and cosmetic procedure-related necrosis (such as post rhinoplasty complications, dermal fillers or nasal botulinum toxin injections side effects…etc), as well as articles not available in English. Any study centered around surgery and/or cosmetology procedure induced necrosis was excluded, systematic reviews were also not eligible for our study. However, studies without accessible full text with abstract available, were analyzed and included, all included studies are listed in the reference Sects [1–39].

### Search strategy

A systematic Search was conducted across 3 databases, PubMed, Scopus, ScienceDirect, from inception to 7 December 2025. The search strategy combined controlled vocabulary (MESH terms) and free-text terms related to nasal necrosis, the full Boolean string applied for PubMed was (“nasal necrosis” OR “cutaneous necrosis of the nose” OR “skin necrosis” OR “nose” OR “nasal” OR “nose tip necrosis” OR “nasal tip necrosis” OR “necrotic rhinitis” OR “nasal gangrene” OR “nasal tissue necrosis” OR “necrotizing facial infection” OR “nasal ulceration” OR “nasal ulcer” OR “nasal ischemia” OR “septal necrosis” OR “nasal sloughing” OR “nasal tip tissue necrosis” OR “nasal necrotizing lesion” OR “destruction of nasal tissue” OR “necrosis of nasal cavity” OR “nasal gangrenous lesions” OR “nasal tissue death” OR “centro-facial tissue necrosis”) AND (“case report” OR “case reports”).

For Scopus and ScienceDirect, the same core terms were adapted to the syntax of each database and combined with filters restricting results to case reports and case series when available.

### Study selection

All records were imported into RAYYAN and duplicates were removed. Two reviewers independently screened titles and abstracts using the predefined PICOS criteria and conflicts were resolved by a third reviewer. Studies deemed potentially relevant were assessed in full text. Articles describing surgical or cosmetic procedure related necrosis, non-human studies, non-English publications, and records without accessible full text were excluded.

### Data extraction and synthesis

The extracted cases were analyzed and segregated into categories according to the patient demographics, presentation, signs and symptoms, laboratory and imaging findings, underlying etiology (congenital, mechanical, infectious, auto-immune, neoplastic and drug induced), as well as non-surgical management and outcome. A table summarizing etiologic categories with corresponding case counts and proportions is presented in Table 1.

### Quality appraisal

Methodological quality of all included cases was assessed using the Joanna Briggs Institute (JBI) Critical appraisal checklists, evaluating each article across eight standardized domains, clarity of patients demographic characteristics, complete patient history and timeline, clear clinical presentation, adequate diagnostic methods and results, clarity of treatment and interventions, report of post-intervention course, documentation of adverse events, and whether the case provided meaningful takeaway. The full quality appraisal results are presented in Supplementary Table 1.

## Results

### Study selection

**Fig. 2:**
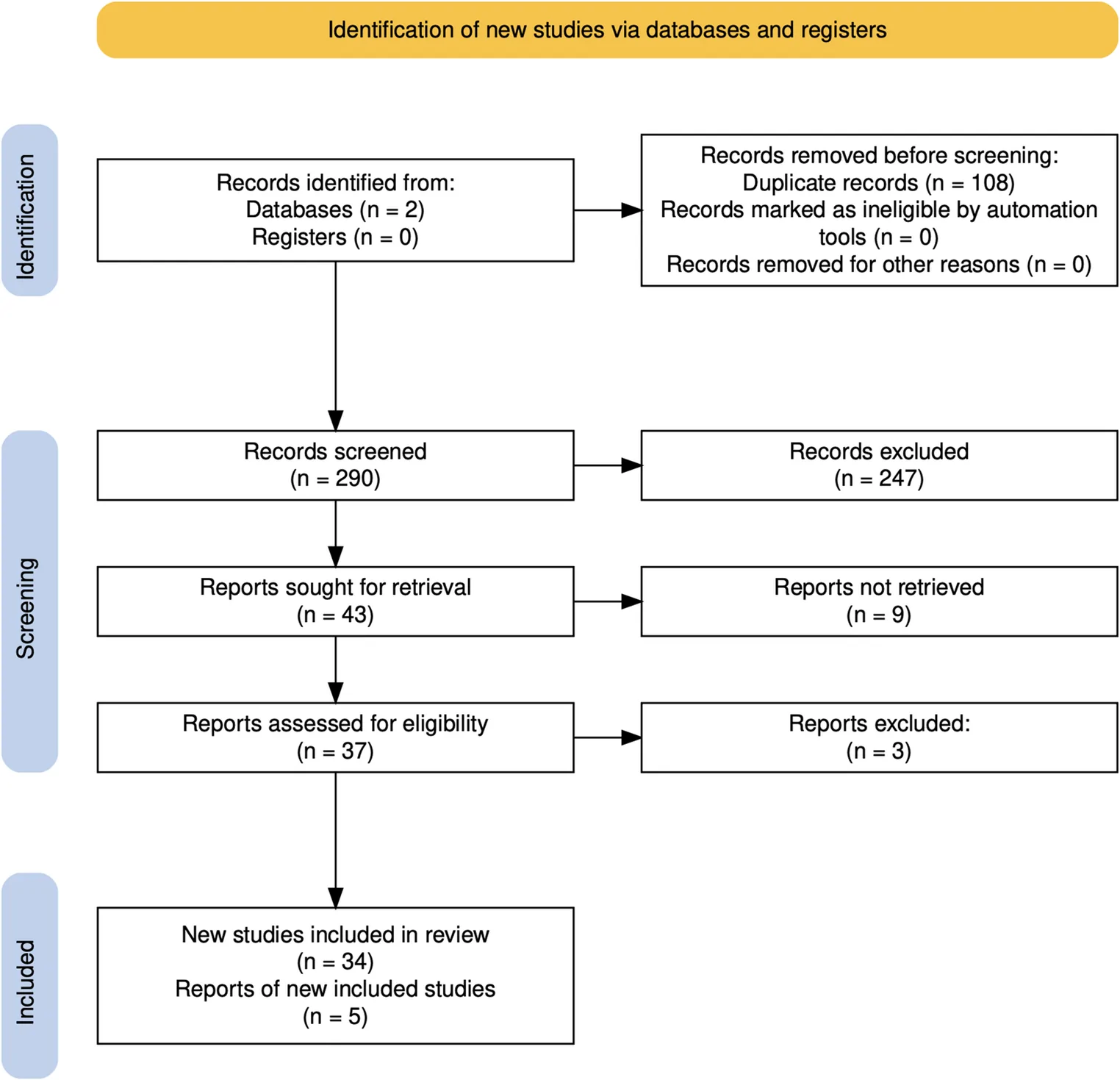
PRISMA Flow diagram of Study Selection

The database search identified 398 records, of which 108 duplicates were removed leaving 290 articles. 256 were excluded after screening for not meeting the inclusion criteria. Full text review was performed for 34 studies, with an additional five case reports identified through manual screening resulting in a total of 39 publications describing 45 cases. A PRISMA 2020 flow diagram summarizing the numbers of records was constructed to ensure transparency of the study selection process (Fig. 2).

### Case characteristics and etiologic distribution

A total of 45 cases were included and categorized based on the underlying etiology of nasal necrosis. Cases were distributed as follows: 2.2% congenital, 6.7% neoplastic, 13.3% immune-mediated (including vasculitic and hypercoagulable states), 13.3% drug-induced (several linked to illicit drug use), 20% mechanical (traumatic events, including those caused by mechanical ventilation), and infectious etiologies were most frequent, 44.4% (viral, bacterial, and fungal). Demographically, most cases occurred in adults (aged 18–86), with an additional five reports focused on pediatric populations. A summary of all etiologic categories with corresponding case counts and proportions is presented in Table 1.

**Table 1:** A summary of all etiologic categories with corresponding case counts and proportions

Etiology	Number of Cases (*n*)	Percentage (%)
Infectious	20	44.4%
Mechanical/Traumatic	9	20.0%
Immune-mediated	6	13.3%
Drug-induced	6	13.3%
Neoplastic	3	6.7%
Congenital	1	2.2%
**Total**	45	100%

### Anatomical extent and sex distribution

Isolated nasal necrosis was observed in 26/45 cases (57.8%). However, 26.7% of patients 12/45 had additional facial involvement, and 15.6% in 7/45 patients showed widespread necrosis affecting multiple body regions. No clear differences were observed between gender in congenital, mechanical, or neoplastic etiologies. In contrast, drug-induced necrosis and immune-mediated causes were more common in females (4 F:2 M and 4 F:1 M, respectively), whereas infectious etiologies were more frequent in males (11 M:7 F).

When analyzing anatomical distribution across etiologies, congenital and mechanical causes were predominantly limited to the nose (consistent with pressure-related mechanisms). Drug-induced necrosis tended to be more widespread, while neoplastic and immune-mediated cases showed no significant distribution patterns. None of the infectious cases presented with widespread necrosis; among them, 12 involved isolated nasal necrosis and 8 were confined to the face.

### Geographic distribution

Notably, most reported cases (33/45, 73.3%) originated from high-income countries. This suggests that additional etiologies of nasal necrosis may remain underrecognized or underreported in lower-resource settings, where such conditions often receive less medical attention and publication coverage.

## Discussion

### Infectious etiologies

#### Bacterial

Among infectious etiologies, bacterial agents play a pivotal role in compromising vessels leading to tissue breakdown. At the top of the list, Pseudomonas putida, usually a rare finding in nasal infections, was reported in necrotizing skin lesions, specifically in immunocompromised patients. One documented case of P. Putida infection was in a patient suffering from liver failure who presented with progressive nasal crusting and necrosis. Even though initial superficial culture swabs tested negative, later tissue biopsy revealed P. Putida indicating the clinical value of deep biopsy when suspicious of necrotizing infections following a negative culture [11].

More frequently encountered in this setting, Pseudomonas aeruginosa is commonly known to cause ecthyma gangrenosum specifically in neutropenic or immunocompromised patients. This hallmark skin lesion starts off as painless macules rapidly progressing to hemorrhagic bullae along with necrosis in the form of black eschars. This condition usually affects the extremities or gluteal region but was notably enough reported as a rare nasal presentation in a neutropenic patient with acute leukemia strongly suggesting its rare capacity to affect the head and neck area [21].

Additionally, Purpura Fulminans has been related to Methicillin-resistant Staphylococcus aureus (MRSA). It’s a severe thrombotic disorder marked by rapidly progressing purpura, disseminated intravascular coagulation, and multi-organ failure. A notable case involved isolated nasal necrosis in a 75-year-old man with underlying myelofibrosis, where blood cultures confirmed MRSA bacteremia as the causative agent while imaging revealed full- thickness necrosis of the nasal dorsum. This progression from initial erythema to extensive infarction despite having underwent antibiotic therapy highlights the aggressive coagulopathic nature and the necessity for early diagnosis and supportive care [2].

Furthermore, Escherichia coli has been documented as a low incidence but potentialy contributes to purpura fulminans with nasal involvement, as reported in a female patient following a septic shock resulting from an unsafe abortion. E.coli was positive in blood cultures and the ongoing DIC caused the skin necrosis of the nasal tip. This case highlights the role of gram-negative sepsis in manifesting thrombotic skin lesions [39].

#### Viral

Although less common than bacterial and fungal triggers, viral infections remain clinically relevant when it comes to nasal necrosis. Among the viruses described in the literature, Varicella-zoster virus (VZV) can rarely but critically present as acute necrosis of the nasal tip. In two cases, patients exhibited painless, hemorrhagic crusted plaques of the nasal tip which were later confirmed by PCR and immunohistochemistry to be linked to VZV. Both cases emphasize the need to take into account VZV in the differential diagnosis of nasal necrosis, even in immunocompetent hosts [19].

In addition to VZV, the Herpes simplex virus (HSV) is more commonly correlated to vesicular lesions but nonetheless may also present with localized necrosis. In such clinical context, immunohistochemistry and PCR are crucial for differentiating HSV from VZV and other causes of necrotic ulceration [19]; the same article reports negative HSV in comparison to VZV positivity.

Finally, a less frequently reported but clinically relevant agent, Chikungunya virus has been reported as a potential cause of necrotic skin lesions of the nasal area amid severe systemic illness. A Venezuelan case series described nasal tip necrosis in patients with virologically confirmed Chikungunya fever who experienced shock and multiple organ dysfunction, suggesting viral-induced vascular compromise [25].

#### Fungal

A variety of fungal pathogens have been implicated in nasal necrosis, each presenting differently as the most aggressive cause of nasal necrosis. Mucormycosis is a devastating fungal infection that is a typical representation in immunocompromised individuals but has also been reported in immunocompetent patients. A case of chronic rhino-orbital mucormycosis in a woman with no significant past medical history resulted in facial necrosis and fatal multiorgan failure, illustrating the destructive potential of mucormycosis even outside traditional risk groups [22].

In clinical presentation, Aspergillosis may mimic mucormycosis but warrants a separate clinical approach. In the same case above, initially, biopsy suggested aspergillosis which led doctors to start the patient on voriconazole. However, postmortem examination confirmed mucormycosis, emphasizing the diagnostic challenges and the imperative for high clinical suspicion and early aggressive treatment [22].

### Immune-mediated etiologies

When evaluating nasal skin necrosis, vasculitic and immune-mediated processes must be considered, as a wide variety of differential diagnoses fall within this category.

For example, giant cell arteritis (GCA) should be suspected in a patient presenting with nasal tip necrosis accompanied by recent ocular symptoms and abnormalities or absence of the temporal artery pulse [1].

A case involving an 83-year-old patient described nasal necrosis secondary to underlying GCA. The patient complained of progressive loss of visual acuity on the left side, and physical examination revealed asymmetric temporal artery pulses as well as retinal abnormalities on fundoscopy. Laboratory findings were consistent with systemic inflammation, including elevated CRP and ESR, and neutrophilia. A temporal artery biopsy confirmed the diagnosis of GCA, while a biopsy of the nasal lesion showed numerous capillary thrombi and extensive epidermal and dermal necrosis. Systemic corticosteroids were the treatment of choice [1].

Evaluation of nasal tip necrosis should include assessment for rheumatologic disease, as it may represent a cutaneous manifestation of rheumatoid arthritis flare [3].

In fact, a case of a 70-year-old female with a past medical history of rheumatoid arthritis presented with a necrotic lesion on the nasal tip accompanied with joint pain and stiffness; histology showed lymphocytic venulitis and conservative debridement led to improvement [3].

Although uncommon, cold agglutinemia should be suspected in cases of diffuse gangrenous skin eruptions following an infection, particularly when necrotic lesions appear in areas with end-arterial circulation, such as the nose tip and distal phalanges.

Upon review of the literature, two cases illustrating this condition were identified, both in female patients aged 12 and 57 years. The lesions evolved from bluish discoloration to necrotic, gangrenous eruptions involving the nose and fingers, as well as the cheeks, ears, and buttocks. Paraclinical investigations revealed characteristic hemolytic anemia and positive anti-I IgM cold agglutinins [4, 27].

Histological findings in cold agglutinin disease showed no evidence of vasculitis, but were instead consistent with thrombotic events [27, 31]. Treatment targets both the underlying cause (e.g., cold agglutinin disease and any associated infection) and the skin manifestations.

Management of the underlying condition may include anticoagulants [31], systemic antibiotics and corticosteroids [27], blood transfusions [27], or even plasmapheresis [31]. Skin lesions are treated with wound care modalities such as hyperbaric oxygen therapy [31], though in severe cases, amputation may be necessary [27].

Cold agglutinin disease can result in nasal tip necrosis even in the absence of preceding infection (i.e., primary cold agglutinin disease). The clinical and paraclinical findings described are consistent with secondary cold agglutinin disease, except for features specific to the infectious trigger [31].

In autoimmune diseases, a thorough clinical investigation is crucial. On one hand, the etiology is not always evident at first sight; on the other, cases can become complicated with overlapping conditions coexisting.

A 23-year-old female presented with a bluish discoloration on her nose tip that progressed to necrosis accompanied by local paresthesia [24]. The patient had several risk factors predisposing her to thrombotic events, including oral contraceptive pills (OCPs) use and smoking. There was no reported recent infection or illicit drug use. On history, she also reported cold exposure.

Extended laboratory workup revealed normocytic normochromic anemia, elevated ESR, complement consumption, and a positive lupus anticoagulant assay. Rheumatoid factor (RF) and cryoglobulins were also positive. In contrast, antinuclear antibodies (ANA), anti-double stranded DNA, extractable nuclear antigen (ENA) panel, and anti-neutrophil cytoplasmic antibody (ANCA) tests were negative. Histology was consistent with thrombosis, and no vasculitis was observed. The final diagnosis was antiphospholipid syndrome overlapping with cryoglobulinemia.This case exemplifies how complex and unexpected the diagnostic process can be. The patient was treated with corticosteroids [24].

### Drug-induced etiologies

Drug-induced eruptions. The most typical presentation is that of levamisole toxicity. Levamisole, previously used for many diseases such as malignancies, rheumatologic conditions, and helminthic infections, was discontinued due to its adverse effects. Among these, the most severe and relevant were hematologic complications (leukopenia, thrombocytopenia). Nowadays, levamisole is essentially found as an adulterant in cocaine. Therefore, illicit drug use must be considered among the differentials when approaching nasal tip necrosis [5].

The skin eruptions induced by levamisole toxicity secondary to cocaine usage range from painful purpuric lesions to full-thickness necrosis [5, 7, 12]. The typical distribution involves the nose, cheeks, and bilateral ears [5]; however, other locations may also be involved, such as the upper and lower extremities and even the buttocks [7], rarely, those lesions can progress and cover up to half of the body surface [12]. Purpura and ecchymosis can be explained by thrombocytopenia, while necrosis is primarily attributed to the thrombotic microangiopathy provoked by the vasculitic reaction secondary to levamisole [5].

For diagnosis, a history of cocaine use along with hemorrhagic lesions in the typical locations is highly suggestive [5, 7, 9, 12]. Tests that help confirm the diagnosis include: neutropenia and thrombocytopenia on CBC [5, 7] positive p-ANCA and c-ANCA [5, 7] (though not universally) [9, 12], urine toxicology for cocaine presence, and skin biopsy showing leukocytoclastic vasculitis with thrombotic microangiopathy [7, 9, 11, 12].

Considering this etiology is associated with drug abuse, patients might present with coexistent bacteremia, hepatitis B virus infection, co-ingested opiates, etc [9]. The mainstay of treatment is, of course, cocaine cessation [5, 7, 9, 12], along with systemic corticosteroids [9], antibiotics (due to the common risk of infection from neutropenia), and necessary supportive care [5, 7, 9, 12]. In extreme cases, excision, debridement, skin grafting [7, 12] or even amputation may also be necessary [12].

Levamisole is certainly not the only drug known to cause nasal tip necrosis; other more commonly used medications have also been reported to produce this cutaneous manifestation. For example, a case of nasal necrosis was documented in a patient just three hours after oral intake of acenocoumarol. The ecchymotic lesions involved the nose as well as the upper and lower extremities. Blood tests revealed signs of inflammation (elevated CRP and ESR), along with increased levels of GGT and ALP. A skin biopsy demonstrated leukocytoclastic vasculitis and paradoxical fibrin thrombi. The treatment of choice involved reversing the anticoagulant effect by administering vitamin K and transfusing the patient with fresh frozen plasma (FFP) [6]. A case of Waldenström’s macroglobulinemia treated with rituximab also resulted in nasal skin and soft tissue necrosis, the lesions recovered spontaneously [13].

### Neoplastic etiologies

Lymphomas are neoplasms of the lymphatic system that can be divided into Hodgkin and non-Hodgkin lymphomas. Non-Hodgkin lymphomas include natural killer (NK) cells, from which extranodal NK/T cell lymphoma of the nasal type is derived [33–35]. Previously, it was known as lethal midline granuloma due to its destructive capacity in the nasal cavity of unknown etiology. The World Health Organization (WHO) then proposed the current name “extranodal NK/T cell lymphoma of the nasal type,” which was adopted by the REAL classification [33, 34]. This lymphoma is rare as it represents 0.44% of extranodal lymphomas of the nasosinusal region [33], however, this neoplasia is characterized by the destruction of soft tissues that affect the structures of the upper respiratory tract: nose, paranasal sinuses, palate and facial soft tissues [33], demonstrated in case reports (Table 2).

**Table 2:** Clinical, histopathological and therapeutic features of extranodal NK/T-cell lymphoma, nasal type (ENKL)

Cancer Type	Case Report	Imaging Findings	Endoscopy	Histopathology/IHC	Differential	Treatment	Outcome	Ref
Extranodal NK/T-Cell Lymphoma, Nasal Type	17 yo, male; Fever (3 d), periorbital edema, purulent rhinorrhea, obstruction	Infiltrative lesion; proptosis; pansinus opacification; nasolacrimal duct involvement	Edema hyperemia, purulent discharge	Initial biopsy: EBV+. Second biopsy: ENKL confirmed	Chronic rhinosinusitis	Radiotherapy + systemic chemotherapy; low-dose steroids	Progressed to pancytopenia, hepatorenal failure; death at 17 days	[33]
	86 yo, female; Chronic rhinosinusitis, obstruction, facial pain, proptosis, fever	Complete opacification of left sinuses; lamina papyracea destruction	Granulomatous obstructing lesion	First biopsy: fungal elements. Second biopsy: ENKL confirmed	Chronic rhinosinusitis	Antifungals + debridement + chemotherapy	Partial response; death at 5 months	
	43 yo, male; 2 weeks nasal pain, swelling, necrotic mass	Necrotic mass eroding lateral nasal wall and ethmoid; septal extension	Ulcerated necrotic mass	Initial biopsy: granulomatous inflammation. IHC: CD30+, CD56+. Repeat biopsy: ENKL	Initially treated as furuncle	Systemic oncologic therapy	Not reported	[34]
Pediatric Nasal NK/T-cell lymphoma	13 yo, male; Aggressive necrotizing midfacial lesion involving nose, lip, cheek, eyelid	Not performed	Not performed	PEH, EBV+. IHC: CD3, CD56, Granzyme-B, Ki-67 ~ 80%	Misdiagnosed as sebaceous carcinoma	Chemoradiotherapy (RT-2/3 DeVIC regimen)	Complete remission at 1 year (with residual morbidity)	[35]

Legend: *ENKL* Extranodal NK/T-cell lymphoma, nasal type, *EBV* Epstein–Barr virus, *IHC* Immunohistochemistry LDH   Lactate dehydrogenase, RT-2/3 DeVIC #x2009;radiotherapy combined with chemotherapy protocol including dexamethasone, etoposide (VP16), ifosfamide, and carboplatin

This type of lymphoma should be considered when there is an ulcerative cutaneous lesion with thickening of the underlying mucosa in the nasal cavity. More than one case has shown that, apart from these characteristic lesions, it can be accompanied by other symptoms such as chronic rhinitis, nasal obstruction and discharge, and pain [33, 34]. When confirmed with imaging studies, there may even be destruction of the lateral nasal wall and the nasal septum [33, 34]. Among its etiopathogenic factors, there is the Epstein-Barr virus, which was positive in two of the cases presented [33, 35], because its oncogenic proteins induce cell proliferation and resistance to apoptosis [33–35].

On the other hand, this type of lymphoma can affect children and adolescents, although it is rare. However, a 17-year-old adolescent with massive edema and necrosis in the midface was reported. A biopsy showed patterns of pseudoepitheliomatous hyperplasia (PEH) in addition to multilobular epithelioid carcinoma resembling carcinoma [34]. Cutaneous carcinoma should be considered as a differential diagnosis. While the frequency of PEH in ENKTCL-NT is 3.8% in adults, only four cases of this lymphoma with PEH have been reported in pediatrics, making this case extremely rare [34].

Diagnosis requires histopathological examination and, occasionally, culture collection. Diffuse infiltrates with an angiocentric and angiodestructive pattern are present, which may accompany areas of necrosis [33, 34]. The neoplastic cells usually express CD2, CD3, CD56, and CD30 in immunohistochemistry, as evidenced in the cases [33, 34].

### Traumatic etiologies

#### Direct trauma

*Trigeminal trophic syndrome (self-inflicted neurogenic ulcers)*.

In this clinical case, progressive ulceration of the right nasal wing in a patient with a history of trigeminal rhizotomy reflects the typical manifestations of trigeminal trophic syndrome (TTS), a rare but significant traumatic cause of nasal necrosis [14]. Unlike other etiologies such as vasculitis, neoplasia or granulomatous infections, TTS presents a characteristic triad: trigeminal anesthesia, burning paresthesias and crescent-shaped ulceration in the affected sensory territory, with preservation of the nasal tip due to its innervation by the anterior ethmoidal nerve [14]. In this context, nasal necrosis is not a consequence of primary vascular ischemia, but of self-inflicted trauma, usually unconscious, triggered by chronic dysesthesias [14]. The absence of specific histological findings and the patient’s initial refusal of manipulation make the diagnosis difficult, making imaging tests, biopsies, autoimmune serologies and microbiological studies essential to exclude infectious, autoimmune or malignant causes [14]. Furthermore, it is important to highlight that the preservation of the nasal tip, innervated by the anterior ethmoidal nerve, was key to direct the diagnosis towards TTS, differentiating it from other causes such as vasculitis (e.g. granulomatosis with polyangiitis), neoplasias or granulomatous infections. The presence of antinuclear antibodies and symptoms compatible with borderline systemic lupus erythematosus further complicated the differential diagnosis, emphasizing the need for a thorough clinical and neurological evaluation [14]. 

#### Pressure-related

*Pressure-related necrosis in neonates (unspecified)*.

In Neonates, the traumatic etiology related to pressure is strongly related to be an in utero pressure-related hypoxic injury of the nasal tip [16]. Skin findings start as erythema and purpura are present at the time of birth before any resuscitation measures. With time, these lesions ulcerate and evolve forming a thick eschar due to superficial necrosis. The pathogenesis is parallelled to halo scalp ring (HSR), in which prolonged pressure exerted during or before delivery produced focal hypoxic-ischemic tissue damage [16]. A number of perinatal risk factors often identified such as caput succedaneum, and conditions like maternal hypertension and preeclampsia. Although the lesions usually heal with conservative management, it often heals with residual scarring highlighting the need for early recognition and appropriate care [16].

### Iatrogenic etiologies

#### Mechanical devices

Nasal injuries related to respiratory devices are a significant complication in both neonates and adults. The skin lesions are usually minor; however, their severity may prevent continued use of noninvasive mechanical ventilation [10, 15, 17, 20]. In most cases, these lesions usually appear after 72 h of continuous exposure, depending on the population and the device used (Table 3).

**Table 3:** Iatrogenic/mechanical nasal tip pressure injuries associated with respiratory support devices

Ventilation mode/interface	Patient & indication	Exposure (h)	Lesion (site & description)	Preventive measures	Ulcer management	Outcome	Ref
HFNC (nasal prongs)	Male, preterm 27 + 3 w; RDS	240	Columella & caudal septum pressure injury with black crusts/necrosis	Not reported	Off-loading bandages between prongs & skin	Not reported	[23]
	Female, preterm 29 w; RDS	120	Nasal tip ulcer with erythema & necrosis	Not reported	Petrolatum + mupirocin	Not reported	
CPAP (full face mask)	Female, 60 y; COPD exacerbation	132	Nasal bridge pressure ulcer	Not reported	Alternative mask; fusidic acid gel	Cardiorespiratory arrest 10 days later; death	[20, 22]
	Male, 68 y; COPD exacerbation, thoracic sepsis, AKI, pulmonary edema	216	Nasal bridge pressure ulcer	Not reported	Not reported	Not reported	
BiPAP (oronasal mask)	Female, 67 y; post-Whipple, sepsis (K. pneumoniae), type II hypercapnic respiratory failure	72	Nasal bridge pressure ulcer	Not reported	Not reported	Not reported	[22]
Nasal PSV (NIV), alternating masks (Profile Lite^®^,Mirage^®^); laser helmet	Male, 19 y; Duchenne muscular dystrophy; septic shock, acute respiratory failure	96	Nasal pressure ulcer from NIV masks	Hydrocolloid dressing; alternating masks; correct strap tension	Multiple interfaces; helmet poorly tolerated → switched to BiPAP with synchrony adjustment; healed after 8 days	Ulcer healed; resumed nocturnal nasal NIV; discharged home	[21]
Nasal PSV (NIV), Alternating masks; later helmet	Male, 18 y; Duchenne muscular dystrophy; coma from decompensated respiratory acidosis	72	Nasal pressure ulcer from NIV masks	Hydrocolloid dressing; alternating masks	Trial of helmet NIV ineffective → switched to BiPAP (RR 18/min, TI 1.3 s); healed after 6 days	Ulcer healed; resumed nocturnal nasal NIV; later died (abdominal sepsis, non-respiratory)	

Lesions are medical device–related pressure injuries of the nasal bridge/tip/columella. Brand names are reported exactly as in the source reports to allow reproducibility. *NR* not reported, *BiPAP* bilevel positive airway pressure, *CPAP* continuous positive airway pressure, *DMD* Duchenne muscular dystrophy, *NIV * non-invasive ventilation, *PSV*  pressure-support ventilation, *RR* respiratory rate, *TI*  inspiratory time

Although most pressure injuries to the nose are preventable, a 42.5% incidence rate of these injuries occurs secondary to the use of nasal cannulas [20], which is attributed to design flaws and a lack of cannula size options for premature infants [20]. In the cases presented, nasal injuries or necrosis occur due to prolonged mechanical pressure exerted by the cannula on the soft tissue, causing focal deformation near the contact site. Because the nasal projections are not flat, the columella protrudes, and the nasal cavities converge inward, causing more pressure than normal, thus favoring these injuries [20]. On the other hand, adults with chronic lung disease are difficult to wean from mechanical ventilators, leading to prolonged use of these devices. In the case of CPAP, patients used it for more than 100 h, which caused the formation of pressure ulcers on the nasal bridge. This is attributed to poorly fitted masks and continuous use for long periods exceeding eight hours that can contribute to continued pressure on the nasal soft tissues [10, 17]. When using BIPAP, infections are considered an important risk factor as they increase the likelihood of necrosis formation [17]. Finally, the constant use of NIV masks with a nasal mask (N-PSV) is necessary in patients with DM, since the continuous friction component due to their high ventilator dependence increases complications related to the nasal mask, in this case, nasal pressure sores [15]. It is important to emphasize that the severity of ventilator dependence has a greater influence on the development of these nasal lesions than age or underlying disease.

One of the best explained mechanisms for the appearance of these lesions in all cases is that when soft tissues (at nasal level) are subjected to a rigid surface (cannula or mask) that generates compression forces and the excess pressure collapses the capillaries producing a reduction in blood perfusion, lymphatic drainage and other microvascular alterations that result in progressive ischemia, thus leading to necrosis [10, 15, 17, 20]. In addition, the humidity plus the heat existing under the mask causes skin maceration causing irritation and increasing the risk of wounds and bacterial or fungal infections [10, 15, 17, 20]. Recommended preventive measures include customizing the device by alternating sizes and adjusting the mask to fit the patient [10, 15, 17]. The use of hydrocolloid dressings or silicone foam may promote healing; however, once the injury is established, pressure should be removed immediately and local healing must be done [10, 15, 17, 20] . 

#### Chemical exposure

- Sodium azide (COVID-19 test kit misuse).

Iatrogenic causes include chemical exposure. In the described case, a patient developed facial skin necrosis, particularly in the nasal region, after incorrect use of a rapid antigen test kit for COVID-19 [8]. The patient reversed the recommended procedure by immersing the swab in the reagent before introducing it into the nasal mucosa, which led to a localized toxic reaction highly likely attributable to the sodium azide contained in the reagent [8]. This substance can induce oxidative stress, inhibition of mitochondrial respiration, and direct tissue damage. Clinically, the condition presented with erythema, edema, ulcerative lesions, and progressive necrosis of the nasal area, findings consistent with the characteristic signs of nasal tip necrosis [8]. In this case, negative cultures, the absence of findings in the nasal mucosa and the temporal correlation with exposure to the reagent allowed to rule out other etiologies, guiding the management towards anti-inflammatory treatment and support of the affected skin [8]. It is important to highlight that necrosis began exactly 48 h after the patient impregnated the swab with the reagent containing sodium azide and introduced it into the nasal mucosa; this short time interval directly links chemical exposure with the appearance of nasal tip necrosis, evidencing that the toxic substance was the immediate trigger of vascular and tissue damage in the nasal apex [8].

### Limitations

This review has several important limitations. First, although the search was thorough, the available literature on nasal necrosis is confined to case reports and small case series, which are prone to selection and publication bias and do not allow causal inference or robust comparison of treatment strategies. Second, our inclusion criteria were restricted to English-language publications, and some potentially relevant articles were inaccessible, which may have introduced language and selection bias and likely underestimates the burden of nasal necrosis in low- and middle-income settings. Third, clinical reporting was heterogeneous: key variables such as detailed diagnostic workup, wound care protocols, and long-term outcomes were often incompletely described, limiting our ability to synthesize standardized management recommendations. Despite these limitations, this review provides the most comprehensive synthesis to date of non-surgical etiologies and management of nasal necrosis and offers a valuable foundation for future clinical research.

## Conclusion

Nasal necrosis presents as a complex clinical entity with several etiologies ranging from fulminant infection and immune mediation to drug induced vasculitis, lymphoma and device related, making its management by dermatologists extremely diverse depending on the cause. This study highlights the importance of early diagnosis, recognition in tailoring the effective treatment with the timely initiation of non surgical management can be organ saving and sometimes lifesaving. By systematically synthesizing 45 cases, this review serves as a foundational initiative towards the development of an evidence-based clinical guideline, where it delineates the principal etiologic categories, characteristic clinical patterns and medical treatment strategies currently reported. These findings provide a practical foundation for diagnostic reasoning, noting that further studies with a bigger cohort and standardized outcome is necessary to refine treatment approaches even more and reach better clinical decision making in this rare condition. 
